# Visualising apoptosis in live zebrafish using fluorescence lifetime imaging with optical projection tomography to map FRET biosensor activity in space and time

**DOI:** 10.1002/jbio.201500258

**Published:** 2016-01-11

**Authors:** Natalie Andrews, Marie‐Christine Ramel, Sunil Kumar, Yuriy Alexandrov, Douglas J. Kelly, Sean C. Warren, Louise Kerry, Nicola Lockwood, Antonina Frolov, Paul Frankel, Laurence Bugeon, James McGinty, Margaret J. Dallman, Paul M. W. French

**Affiliations:** ^1^ Institute of Chemical Biology, Department of ChemistryImperial College LondonSW7 2AZUK; ^2^ Department of Life Sciences, Imperial College LondonSW7 2AZUK; ^3^ Photonics Group, Department of Physics, Prince Consort RoadImperial College LondonSW7 2AZUK; ^4^ Division of MedicineUniversity College LondonGower StreetLondonWC1E 6BTUK; ^5^ COMPLEXUniversity College LondonGower StreetLondonWC1E 6BTUK

**Keywords:** OPT, FLIM, FRET, zebrafish, Caspase 3

## Abstract

Fluorescence lifetime imaging (FLIM) combined with optical projection tomography (OPT) has the potential to map Förster resonant energy transfer (FRET) readouts in space and time in intact transparent or near transparent live organisms such as zebrafish larvae, thereby providing a means to visualise cell signalling processes in their physiological context. Here the first application of FLIM OPT to read out biological function in live transgenic zebrafish larvae using a genetically expressed FRET biosensor is reported. Apoptosis, or programmed cell death, is mapped in 3‐D by imaging the activity of a FRET biosensor that is cleaved by Caspase 3, which is a key effector of apoptosis. Although apoptosis is a naturally occurring process during development, it can also be triggered in a variety of ways, including through gamma irradiation. FLIM OPT is shown here to enable apoptosis to be monitored over time, in live zebrafish larvae via changes in Caspase 3 activation following gamma irradiation at 24 hours post fertilisation. Significant apoptosis was observed at 3.5 hours post irradiation, predominantly in the head region.

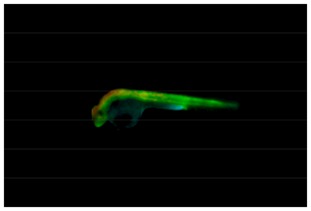

## 1 Introduction

Microscopy of cells in culture has been invaluable in biology, leading to countless discoveries of cellular function, cell‐cell communication, protein‐protein interactions and protein structure. There is, however, an increasing drive to image functional cell behaviour in the context of whole live organisms to enable the study of systemic effects of disease and potential therapies as well as to further the understanding of localization and propagation of signalling responses 1, 2, 3.

Zebrafish are well suited to whole‐body imaging since they are relatively transparent to optical radiation and are genetically tractable, with transparent mutants and fluorophore‐tagged lines readily available. 3‐D imaging technology that is suitable for zebrafish, however, is still under development with commercial *in vivo* imaging platforms mainly providing intravital microscopy within larger specimens 4 or lower magnification wide‐field imaging of whole fish with no 3‐D information. One approach is to combine PET/MRI/CT scans with laser scanning confocal microscopy (LSCM) to provide sufficient resolution for the detection of tumor volume and metastasis 5, but this cannot enable the functional activation of proteins to be visualised throughout a live fish. Micron‐scale computed tomography (Micro‐CT) is a powerful tool for imaging small specimens and is being used as the primary tool in the Zebrafish Phenome project 6. It can provide cellular resolution through the use of highly coherent, synchrotron generated X‐rays, either alone for imaging of bone or in conjunction with contrast agents for soft tissue imaging. However, this approach requires samples to be fixed so cannot be applied *in vivo* and probe development is not yet at the stage to permit monitoring of functional protein activity 6, 7. Accordingly there is much interest in developing “mesoscopic” optical imaging techniques for 3‐D imaging of live organisms such as zebrafish. These include optical projection tomography (OPT) 8, which can be simply implemented as wide‐field imaging applied to a rotating sample, and light sheet microscopy (LSM) 9, 10, 11, which combines illumination with a focussed light sheet and orthogonal or oblique wide‐field fluorescence imaging with scanning of sample or light sheet. We choose to work with OPT but note that LSM techniques can also be applied to image zebrafish embryos 12, 13, 14 although they typically have a sub‐mm field of view and therefore require multiple acquisitions for larger specimens.

OPT is analogous to x‐ray CT, but uses visible optical radiation. It can be implemented to image with transmitted light and with fluorescence, and so can utilise a vast array of fluorescent markers and biosensors. Because the resolution of OPT does not rely on the focussing of the illuminating radiation, it is easily scaled over a range of sample sizes and is relatively insensitive to moderate scattering, requiring only adequate transmission of the illuminating and transmitted/emitted radiation to image at greater depths. Because it is a wide‐field illumination and detection technique, the incident light intensities associated with OPT are lower than those associated with laser scanning microscopy, potentially reducing nonlinear contributions to photobleaching/phototoxicity, and OPT is able to image entire mesoscopic samples in a few minutes, which is important for live animal imaging when the sample is anaesthetised. These advantages have led to OPT techniques being developed to provide increased sensitivity 15, higher resolution 16, faster acquisition times 17, 18 and increased spectroscopic content, including blood flow 19, high dynamic range imaging [20] and FLIM, which we have previously demonstrated with OPT 21 and applied to whole body *in vivo* imaging of zebrafish embryos 22.

Here we report the application of OPT to map the activity of a Förster resonant energy transfer (FRET) biosensor throughout a whole intact zebrafish using FLIM. FRET essentially reads out the colocalisation (≲10 nm) of two or more fluorophores via the strength of the resonant energy transfer that occurs between them 23. This energy transfer can be read out in many ways 24 but in live samples is most commonly done by detecting or measuring the change in the ratio of “donor” fluorophore to “acceptor” fluorophore emission (spectral ratiometric FRET) or by detecting the change in the fluorescence lifetime of the donor, both of which decrease with increasing FRET efficiency.

**Figure 1:**
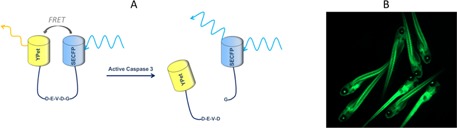
The Caspase 3 FRET biosensor design and its expression in transgenic fish. (A) In the Caspase 3 FRET biosensor, SECFP and Ypet are linked with a flexible 23 amino acid chain, which includes the Caspase 3 cleavage sequence DEVDG in the centre. Upon cleavage of this site in the presence of Caspase3, the SECFP and Ypet become unlinked and the fluorescence lifetime increases. (B) Tg(Ubi : Caspase3bios) zebrafish were generated using the TOL2 Gateway system. Founder fish were imaged at 5 dpf, using a GFP filter cube to assess transgene expression levels.

Spectral ratiometric FRET depends on relative sensitivity of the donor and acceptor spectral channels and the relative attenuation of these signals and so needs to be calibrated for each instrument and can change from sample to sample. It is also subject to artefacts resulting from spectral cross‐talk that can produce severe artefacts if the stoichiometry of donor and acceptor concentrations is unknown 25. The latter issue can be mitigated by using single chain FRET biosensors that have a fixed stoichiometry. Essentially FRET biosensors are a single molecule construct incorporating both donor and acceptor fluorophores that change their conformation or are cleaved upon interaction with their analyte such that the donor‐acceptor distance and therefore the FRET efficiency is modified. The Caspase 3 FRET biosensor applied in this study is based on a previously published Caspase 3 FRET biosensor 26. It is designed to be cleaved by active Caspase 3 such that the donor and acceptor become separated by more than ∼10 nm and the FRET efficiency goes to zero. This is illustrated in Figure 1. FRET of protein activity in live zebrafish embryos has previously been demonstrated using spectral ratiometric readouts of single chain FRET biosensors in a wide‐field microscope 27, 28 and using confocal microscopy 29, 30.

FLIM is widely considered to be a more robust readout of FRET 31, 32, 33 since it requires only measurement of the donor fluorescence, thereby avoiding problems associated with the inner filter effect and spectral crosstalk between donor and acceptor channels and allowing measurements to be compared between different instruments and samples without calibration. Fluorescence lifetime measurements are inherently ratiometric and therefore also independent of fluorophore concentration and excitation/detection efficiencies, making them relatively robust for measurements in scattering media such as biological tissue. FLIM 34 can be implemented with wide‐field detection using time‐gated 35 or modulated 36 imaging detectors and time‐gated imaging has recently been combined with LSM 37. It can also be implemented in laser scanning confocal 38 or multiphoton 39 microscopes, typically implemented using time‐correlated single photon counting (TCSPC) detection 40.

FLIM of FRET biosensors expressed in live zebrafish have been read out using laser scanning multiphoton microscopy 41, 42 with FLIM implemented using TCSPC. While this can provide high resolution FLIM over a small volume, it would be challenging to apply multiphoton TCSPC FLIM to map activity of a FRET biosensor throughout a whole zebrafish embryo – partly due to the relatively small field of view and long acquisition times associated with multiphoton microscopy, which are challenging if the sample is to be anaesthetized, and partly due to the phototoxicity that would result from the prolonged exposure to intense radiation. We therefore choose to implement FLIM OPT with wide‐field time‐gated detection, which requires acquisition times as short as a few minutes to image a whole zebrafish embryo, and for which we have previously shown the phototoxicity is low enough to enable *in vivo* imaging of live Tg(lysC : GFP) embryos, in which myeloid cells were labelled with GFP 22.

Here we apply FLIM OPT to map the activity of a FRET biosensor that is cleaved by Caspase 3, resulting in an increase of the donor fluorescence lifetime 43. Caspase 3 is a member of the cysteine dependent aspartate proteases, a family of proteases that cleave specific peptide sequences after an aspartate residue. It is a key enzyme in apoptosis, or programmed cell death, which is essential during development and homeostatic tissue turnover as well as during disease. If apoptosis becomes dysregulated it can lead to cancer, autoimmune disorders and neurodegenerative diseases 44, 45. Caspase 3 FRET biosensor probes have previously been used in live cells in culture and in drosophila 3, 46, 47, 48, 49, 50. It is interesting to study Caspase 3 in higher organisms that can serve as more realistic models for human disease, but transgenic mice stably expressing functional FRET probes are difficult to generate and very few have been reported, although Yamaguchi et al. succeeded in creating and imaging transgenic Caspase 3 FRET biosensor mice using confocal intensity imaging of the embryo 51. There are however reports of zebrafish with FRET probe transgenes, imaged using LSCM 26, 27, 28, 52. We have constructed a Caspase 3 FRET biosensor and generated fish in which this transgene is controlled by the Ubiquitin promoter (Tg(Ubi : Caspase3bios)). We tested the biosensor's activity in a model of gamma irradiation, which is known to cause localised apoptosis in the embryo 53. In this paper we demonstrate, we believe for the first time, that FLIM OPT can map changes in the FRET signal of a Caspase 3 biosensor following gamma irradiation, used as a means to induce and follow apoptosis, in whole live Tg(Ubi : Caspase3bios) zebrafish embryos. FLIM OPT was complemented by laser scanning confocal FLIM microscopy in order to optimise the protocol for labelling and irradiating the zebrafish embryos and validate the FLIM OPT readout of FRET.

## 2 Results and discussion

In order to demonstrate *in vivo* FLIM readouts of the Caspase 3 FRET biosensor throughout whole intact zebrafish embryos, it was necessary to address three main challenges: (i) the generation and validation of appropriate transgenic zebrafish expressing the Caspase 3 biosensor; (ii) the optimisation of OPT imaging instrumentation and protocols for live zebrafish embryos and (iii) the analysis of the large, complex FLIM OPT data sets to realise the quantitative FRET readouts.

To address the first challenge, we developed constructs for ubiquitous expression of the Caspase 3 FRET biosensor in zebrafish, shown in Figure 1A and as discussed in Materials and Methods. We also developed a construct for ubiquitous expression of just the SECFP donor in zebrafish to provide a control for non‐specific variations in donor lifetime. These constructs were first tested in mammalian (HeLa) cell culture using an automated multiwell plate FLIM microscope, as discussed in the Supplementary Information, and reference lifetimes for SECFP and the biosensor undergoing FRET were determined (Figure S1).

Transgenic zebrafish expressing these constructs were then generated on the TraNac background which have a double mutation in the *transparent* and *nacre* genes, resulting in a deficit in melanocyte and iridophore formation and are thus relatively transparent to optical wavelengths, particularly during larval stages. They do increasingly scatter optical radiation as they develop into adulthood, although they are still much less opaque than wild‐type fish. The transgene for Tg(Ubi : Caspase3bios) fish included the Ubiquitin promoter upstream of the SECFP ORF, the Caspase 3 specific cleavage site DEVDG, which is included in a flexible linker chain and the YPet ORF. SECFP is a ’super enhanced' variant of CFP, which is more stable to pH changes 54. Transgenic zebrafish embryos produced using this construct ubiquitously and brightly express the Caspase 3 biosensor under excitation wavelengths of 405–525 nm (Figure 1B).

To validate correct expression and functioning of the expressed Caspase 3 biosensor, we undertook FLIM, implemented with TCSPC in a LSCM. We also confirmed the FRET readout of the Caspase 3 biosensor using spectral ratiometric imaging (as shown in Figure S3). To activate the Caspase 3 biosensor in a controlled manner, we induced apoptosis using gamma irradiation. It has previously been shown that irradiation of 24 hours post fertilization (hpf) zebrafish embryos with 15 Gy induces apoptosis that could be visualised *ex vivo* in the head at 3 hours post irradiation (hpi) by staining with an active Caspase 3 antibody 53. The minimum lethal dose for adult zebrafish has been reported as 40 Gy, causing death after 14 days 55. The same report demonstrated the use of 20 Gy as a sub‐lethal dose to ablate lymphocytes, with the fish surviving for a number of months afterwards. To provide a convenient means to reproducibly induce apoptosis, we irradiated zebrafish embryos with 18 or 25 Gy and observed that they survived with no abnormalities until 5 days post fertilisation (data not shown).

**Figure 2:**
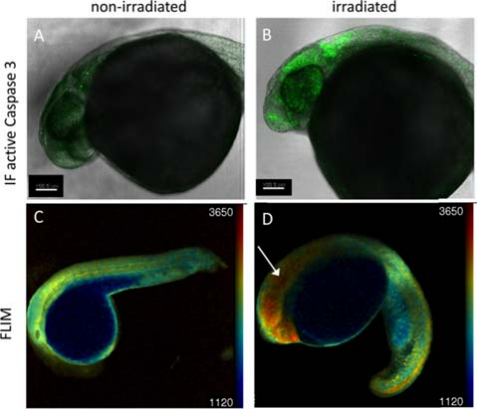
Validation of Caspase 3 biosensor activation following gamma irradiation using confocal microscopy. 24 hpf zebrafish embryos were irradiated (**B**, **D**) with 18 Gy from a ^137^Cs source or left untreated (**A**, **C**) and imaged at 3 hpi using confocal microscopy. (**A**, **B**) confocal fluorescence intensity images of TraNac embryos, either non‐irradiated (**A**) or irradiated (**B**), fixed and labelled with an active Caspase 3 immunofluorescent antibody. (**C**, **D**) confocal fluorescence lifetime images of non‐irradiated (**C**) and irradiated (**D**) Tg(Ubi : Caspase3bios) embryos at 3 hpi.

In this validation study, a proportion of 24 hpf TraNac embryos were irradiated with 18 Gy, fixed at 3 hpi and along with non‐irradiated embryos of the same age, were stained using the active Caspase 3 antibody and imaged using fluorescence LSCM. Figure 2B shows that, at 3 hpi, there is localized Caspase 3 activity in the head of the fish, which is not apparent in non‐irradiated fish of Figure 2A. The experiment was then performed with live Tg(Ubi : Caspase3bios) zebrafish embryos. At 24 hpf, embryos were irradiated, anaesthetized and mounted in agarose for *in vivo* FLIM using LSCM with TCSPC to image a single optical section through the zebrafish embryo. The lifetime data was analysed using locally developed software (*FLIMfit* 56, written in MATLAB) fitting the SECFP donor fluorescence decay profiles pixel‐wise to a single exponential decay model. Although SECFP presents a multi‐exponential decay profile 57, fitting to a single exponential decay model provides a quantitative means to map variations in the response of the FRET biosensor and requires fewer detected photons for robust fitting, thereby reducing the acquisition time and light dose. Lifetime maps were merged with intensity images to suppress regions of low intensity where there was negligible probe expression. As can be seen in Figure 2, FLIM can provide a visualisation of the distribution of Caspase 3 activity, with a longer donor lifetime indicating Caspase 3 activity as the biosensor is cleaved by Caspase 3 and FRET is terminated. We note that measurements of the fluorescence lifetime of SECFP expressed in HeLa cells in culture resulted in a mean lifetime of between 1800–2000 ps for a fit to a single exponential decay model (Figure S1), which agrees with what we observe in Figure 2D where we assume Caspase 3 has been activated and therefore the FRET biosensor is cleaved. This Caspase 3 activation is seen to be localized at the head region, in agreement with the Caspase 3 immunofluorescence image of Figure 2B. Compared to immunofluorescence imaging, FLIM enables the zebrafish embryos to be imaged alive, thereby providing more physiologically relevant information and enabling longitudinal studies or repeat measurements of individual fish, potentially reducing the number of fish required for assays. Time is also saved through not fixing and staining the sample. The LCSM TCSPC image acquisition time for a single confocal optical section was 300 s. While this is much faster than staining protocols, it is too slow to acquire a full 3‐D fluorescence lifetime map throughout the whole zebrafish embryo. The low light dose of OPT and acquisition of a whole sample should result in much faster imaging, allowing for acquisition of whole embryo 3‐D lifetime maps.

The FLIM OPT instrumentation was optimised for imaging live zebrafish embryos mounted in short lengths of vertical translucent FEP tubing and immobilised in 0.7% low melt agarose containing 4.2% MS‐222 as anaesthetic. The zebrafish embryos could be maintained alive for up to at least 6 hours under these conditions (data not shown). The optical set‐up was conceptually similar to that reported in 22 but, as discussed in Materials and Methods, was implemented on a custom‐built wide‐field microscope comprising a tube lens and x4 objective lens configured on a horizontal optical axis with the zebrafish rotating in the FEP tubing around a vertical axis. FLIM was implemented using wide‐field time‐gated imaging such that, for each OPT acquisition, a series of 5 time‐gated fluorescence intensity images were acquired at each projection angle and a filtered back projection (FBP) algorithm was used to reconstruct the 3‐D intensity image corresponding to each time‐gate delay. From these data the mean fluorescence lifetime for each voxel could be calculated using our in‐house software, *FLIMfit* 56, as discussed in Materials and Methods. For this work we acquired FLIM data at 90 projections with an interval of 4° as the sample rotated through 360°. The total acquisition time for each 3‐D data set (comprising 450 time‐gated images) was ∼150 s.

**Figure 3:**
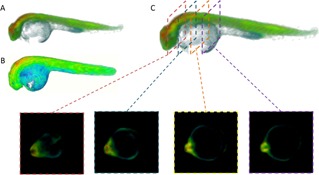
Region of interest selection for data analysis. Rendered 3‐D images of 24 hpf Tg(Ubi : Caspase3bios) zebrafish following 25 Gy gamma irradiation, derived from FBP reconstruction and pixelwise fitting in *FLIMfit* to a single exponential decay model of (**A**) whole FLIM OPT data set, showing intensity merged false colour lifetime (**B**) whole FLIM OPT data set, showing false colour lifetime, without intensity merging, to highlight short lifetime contribution of yolk and (**C**) FLIM OPT data set showing the 2‐D slices through the ROI of the reconstructed fluorescence lifetime image of the segmented dataset.

The analysis of the large FLIM OPT data sets was undertaken using locally developed software tools developed in MATLAB (MATLAB 2014b, The MathWorks Inc.) and Icy [57] (http://icy.bioimageanalysis.org). As can be seen in the Figures 2C and D, the embryo yolk sac presents autofluorescence with a relatively short mean fluorescence lifetime at the SECFP excitation wavelength (∼1200 ps – see supplementary information Figure S2). It was therefore necessary to remove the yolk sac from the reconstructed datasets to avoid contamination of the determination of the mean fluorescence lifetime of the embryo. This was achieved by applying an intensity‐based segmentation algorithm to the last time gate (details given in supplementary information), in which the yolk sac signal is significantly reduced due to its short fluorescence lifetime, and from this generating a binary mask that could be applied in *FLIMfit* so that only voxels in the zebrafish itself were used in the fluorescence lifetime analysis. Figure 3C show an example of a rendered 3‐D FLIM OPT segmented reconstruction derived from only the voxels in the embryo. The programs for reconstructing the OPT data, segmenting the image data sets and calculating the lifetime were configured for automatic batch processing to expedite the data analysis. Further quantitative analysis of the final 3‐D fluorescence lifetime images could then be undertaken using MATLAB or Icy, for example, counting the number of pixels in a region of interest (ROI) volume over a given threshold value was realised using the Intensity Evolution program in Icy. Further details of the data analysis are given in the Materials and Methods section.

**Figure 4:**
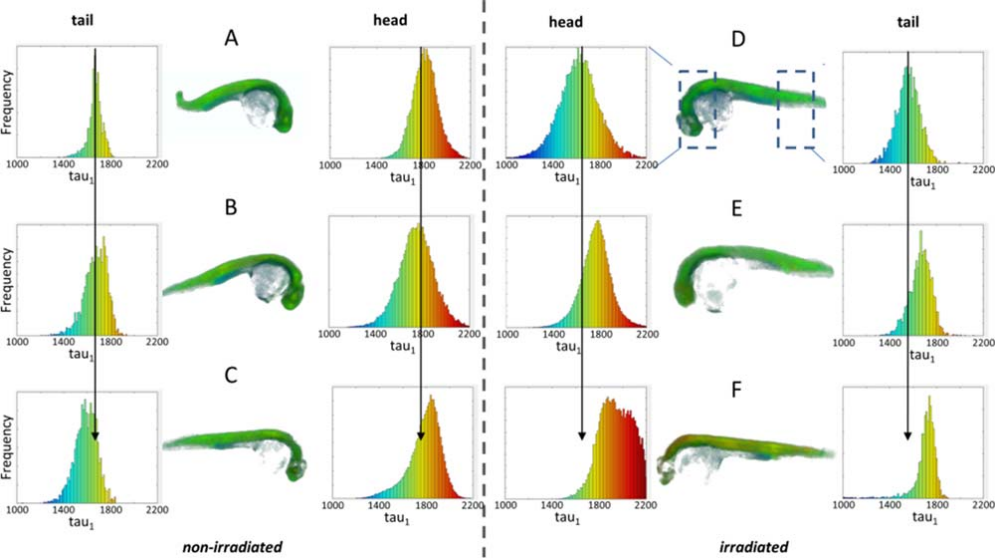
Irradiation causes an increase in fluorescence lifetime at 3.5 hours in the head region of zebrafish. 24 hpf Tg(Ubi : Caspase3bios) were irradiated (**C–F**) with 25 Gy from a ^137^Cs source, or left untreated (**A**–**C**) and imaged at 1 hpi (**A**, **D**), 2 hpi (**B**, **E**) and 3.5 hpi (**C**, **F**) with FLIM OPT. 5 time gated images were acquired every 4°, through a full 360° rotation. Data was reconstructed using MATLAB, and fitted using a single pixelwise variable projection model in FLIMfit, giving the mean lifetime per pixel. 3D images were generated using the 3D viewer plug‐in in FiJi. Histograms show frequency of pixel lifetimes. Centre histograms: head region. Edge histograms: tail region.

FLIM OPT was thus applied to study Caspase 3 activity and localization in 3‐D, reading out the Caspase 3 FRET biosensor in whole live 24 hpf Tg(Ubi:Caspase3bios) zebrafish embryos. Zebrafish were irradiated with 25 Gy, to ensure high levels of apoptosis, and imaged, along with parallel non‐irradiated zebrafish, at set intervals of 1, 2 and 3.5 hours post irradiation (hpi). As shown in Figure 4, the SECFP donor lifetime histograms shift to longer lifetimes at 2 hpi in irradiated fish, indicating decreased FRET and increased Caspase 3 activity, and shift further to longer SECFP lifetimes at 3.5 hpi.

**Figure 5:**
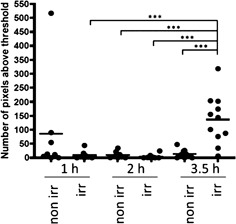
Irradiation causes a statistically significant increase in fluorescence lifetime at 3.5 hours after irradiation. RAW lifetime data was imported into Icy and the ROI Intensity Evolution tool was used to count the number of pixels above a threshold lifetime, within the whole embryo. After testing for normality, data was analysed using a two‐way ANOVA with Bonferroni's post test in GraphPad Prism. Three experiments were pooled, and show a significant difference in pixels above 2 ns lifetime (cleaved probe) ***p < 0.001, in 3.5 hour post irradiation, irradiated fish, compared to all other groups. Each dot represents an individual fish. *n* ≥ 8

If this experiment is repeated with zebrafish expressing SECFP alone, there is no discernible lifetime shift between irradiated and non‐irradiated zebrafish embryos at 3.5 hpi (see supplementary information, Figure S3), indicating that this change in donor lifetime is due to a change in FRET and not in the local fluorophore environment. Figure 4 also indicates that the shift in lifetime histogram is significant in the head region but not in the tail region. This is expected and consistent with published active Caspase 3 immunostaining 50. Indeed, Caspase 3 is strongly expressed in the head region at this stage of embryogenesis as detected by *in situ* hybridisation 58, hence activation of Caspase 3 is expected to be localised in this area. Figure 5 presents a simple statistical analysis showing the variation in the number of pixels that fall above a lifetime of 2 ns (set to select non FRETing SECFP donor fluorescence after cleaving of FRET biosensor).

This shows a statistically significant difference with *p* < 0.001 between irradiated samples at 3.5 hpi versus all other groups imaged, with the exception of 1 hour post irradiation non‐irradiated which has a higher average due to one outlier. It is therefore highly unlikely that the results are due to chance; a statistically significant increase in lifetime is seen in response to gamma irradiation, suggesting the Caspase 3 FRET biosensor is functional. The results reported herein indicate that it is possible to monitor and localize Caspase 3 activity *in vivo* throughout whole intact zebrafish using FLIM OPT to map changes in the emission of the Caspase 3 FRET biosensor.

## 3 Materials and methods

### 3.1 Zebrafish husbandry

Zebrafish lines were maintained according to standard practises and all procedures conformed to UK Home Office requirements (ASPA 1986). Mutant lines used in this study were transparent TraNac (*Tra^–^/Tra^–^; Nacre^–^/Nacre* ^–^ double mutant) with reduced iridophore and melanophore numbers, provided by Julian Lewis at the Cancer Research Institute. Zebrafish life stages are determined by the following ages: embryo stage 0 to 3 days post fertilization (dpf), larval stage 3 to 30 dpf and adult stage aged over 90 dpf. Embryos were maintained in system water and 0.1% methylene blue until imaging.

### 3.2 Design of the FRET biosensor

The FRET biosensor design was based on the previously published SCAT3 26. A 17‐amino acid flexible linker was placed between the two fluorophores, which contained the Caspase 3 consensus cleavage sequence DEVD (final amino acid sequence: SSSELS*DEVD*GTSGSEF).

The peak excitation/emission wavelengths of the donor fluorophore, SECFP, are 438/476 nm and of the acceptor, YPet, are 517/530 nm.

### 3.3 Molecular biology

The Ubi‐Caspase 3 biosensor transgenesis plasmid was generated using the TOL2‐Gateway technology 59. The Caspase 3 biosensor is a single chain biosensor in which a single large open reading frame (ORF) includes the SECFP ORF, followed by a linker containing the DEVDG cleavage site for Caspase 3, and the YPet ORF. The Caspase 3 biosensor was first cloned into the pDONR221 entry clone while the pENTR5′‐Ubi entry clone was described elsewhere 60 and obtained from Addgene (Plasmid #27323). DNA sequences were verified for both entry clones. These were further combined into the pDEST‐TOL2‐pA2 transgenesis vector using Gateway cloning. The Ubi‐SECFP transgenesis plasmid was generated using the same method except that the SECFP ORF was cloned into pDONR221 and sequence verified. The sequences of the final transgenesis constructs: pDEST‐TOL2‐pA2‐Caspase 3 biosensor and pDEST‐TOL2‐pA2‐Ubi‐SECFP are given in the supplementary information.

### 3.4 Zebrafish transgenesis

TOL2‐mediated transgenesis was used to create both Ubi‐Caspase 3 biosensor and Ubi‐SECFP transgenic lines [59, 61, 62]. pDEST‐TOL2‐pA2‐Ubi‐Caspase 3 biosensor or pDEST‐TOL2‐pA2‐Ubi‐SECFP plasmid DNA (*C* = 15 ngs/µL) was co‐ injected with *transposase* mRNA (*C* = 25 ngs/µL) in one‐cell stage TraNac embryos. The injection volume per embryo was approximately 2.5 nL. Injected embryos were raised to adulthood, individually outcrossed and screened for fluorescence to identify founders capable of germline transmission. 1/28 fish screened was a founder for Ubi‐Caspase 3 biosensor while 1/3 fish screened was a founder for Ubi‐SECFP. Identified founders were outcrossed to TraNac to generate stable transparent TraNac transgenic lines. The work described in this paper utilises F2 and F3 embryos.

### 3.5 Sample preparation and mounting

Tg(Ubi : Caspase3bios), Tg(Ubi : SECFP) and TraNac lines were used in the following experiments. Embryos were maintained in system water containing 0.1% methylene blue. For OPT imaging, 24 hpf embryos were immobilised in 0.7% low melt agarose containing 4.2% MS‐222 (Sigma) as anaesthetic. They were drawn in to short lengths of translucent FEP tubing (06406‐60, Cole‐Palmer) with inner and outer diameters of 0.8 and 1.6 mm respectively. For confocal imaging, samples were immobilised in 0.7% low melt agarose containing 4.2% MS‐222 and mounted on a glass coverslip.

### 3.6 Irradiation

Zebrafish embryos were manually dechorinated and placed in 60 mm petri dishes containing system water and 0.1% methylene blue. Dishes were placed in the centre of a ^137^Cesium source irradiator and were dosed with 18 (LSCM) or 25 (OPT) Gy.

### 3.7 Immunofluorescence

31 hpf fixed TraNac embryos that were non‐irradiated or irradiated (18 Gys; 3 hours post‐irradiation) were stained for Active Caspase 3 as per Sorrells et al., 2013 53. The primary antibody was Rabbit anti‐active Caspase 3 (BD Pharmingen Cat. #559565; 1 : 500) and the secondary antibody used was donkey anti‐Rabbit Alexa555 (Life Technologies Cat. #A31572; 1 : 500).

### 3.8 FLIM with LSCM TCSPC

For TCSPC FLIM measurements, a LSCM (TCS SP5, Leica Microsystems GmbH) was used with excitation of SECFP provided by a spectrally‐filtered, (432/24 nm) ultrafast fiber‐laser‐pumped super‐continuum source (SC‐400‐6, Fianium Ltd). The florescence was detected through an emission filter (483/32 nm). A single optical section through each zebrafish embryo was imaged using a 10× objective with a numerical aperture of 0.3, which required an acquisition time of ∼300 s.

### 3.9 FLIM OPT instrumentation

The FLIM OPT set‐up was implemented around an in‐house wide‐field microscope design comprising a ×4 objective (N4X‐PF, Thorlabs Inc) and tube lens (ITL200, Thorlabs Inc) configured to image samples rotating about a vertical axis. The tube‐mounted zebrafish embryos were suspended in a water‐filled cuvette (700‐015‐10, Hellma UK Ltd) to produce a refractive index‐matched environment and to allow stable sample rotation without lateral movement as is required for OPT. The samples were imaged with an aperture positioned directly behind the objective lens to limit the numerical aperture to 0.07. During each OPT acquisition, fluorescence and/or transmitted light images were acquired at 4° intervals as the sample rotated through 360°. A spectrally‐filtered (434/24 nm), ultrafast fiber‐laser‐pumped super‐continuum source (SC‐400‐6, Fianium Ltd) was used to excite SECFP fluorescence in the specimen 63. The emitted fluorescence was imaged via an emission filter transmitting in the range 483/32 nm onto the photocathode of the GOI (HRI, Kentech Instruments Ltd), with the time‐gated image recorded by a CCD camera (Clara, Andor Technology plc). The positions of the time‐gates with respect to the excitation pulses were adjusted to fall between 1500–7000 ps, for example 1500, 3000, 4500, 6000, 7000 ps after excitation, using a computer‐controlled electronic delay line (HDG, Kentech Instruments Ltd) that was synchronized to the excitation laser repetition rate. For each acquisition 5 time‐gated images were recorded at each angular position. The total acquisition time for each data set was ∼2.5 minutes. Each frame had an integration time of 0.05–0.2 s depending on brightness. The GOI has an effective pixel size of ∼26 µm and used in this optical configuration results in an in‐focus lateral spatial resolution of ∼13 µm.

### 3.10 Analysis of FLIM OPT datasets

3‐D image reconstruction was realized using a FBP algorithm in MATLAB. Assuming a parallel projection regime, each horizontal row of pixels in an acquired projection image can be considered a 1‐D projection, i.e. a sum along the direction of the optical axis, of a 2‐D slice through the sample. As the sample rotates these individual 1‐D projections make up the sinogram. From the sinogram, the image signal (here the time‐gated fluorescence intensity) at each point in a 2‐D slice through the sample can be reconstructed using the inverse Radon transform 64. This tomographic reconstruction approach is repeated for each set of time‐gated fluorescence images corresponding to each time delay. In this case, five time‐gated 3‐D intensity reconstructions of the fluorescence signal were obtained. The intensity decay in corresponding voxels of the time‐gated reconstructions was then determined, assuming a single exponential fluorescence decay model, using an in‐house fitting algorithm, *FLIMfit*, which is written in MATLAB and is based on non‐linear least squares optimization and variable projection 65. This can utilise a measured instrument response function and take account of background fluorescence and produces 3‐D reconstructions of both the integrated fluorescence intensity and lifetime. Data was fitted using FLIMfit 65 open source software. A reference measurement of a solution of coumarin 6 dye with a lifetime of 2.5 ns was used to determine the instrument response function (IRF). A time varying background (TVB) map was obtained from a measurement of a well containing media but no cells. The data was then fitted pixel‐wise to a single exponential decay model. For wide‐field time‐gated FLIM data, a separable nonlinear least square fitting algorithm was used and for confocal TCSPC FLIM a maximum likelihood estimation algorithm was used 65. For visualization purposes the lifetime is typically represented on a false colour scale and merged with the integrated fluorescence intensity to suppress noise in regions with little or no signal.

To obtain quantitative information concerning the number of pixels in a ROI with a mean lifetime above a set value (set to 2 ns for Figure 5), the ROI intensity evolution tool in Icy was used with the lifetime images being loaded into Icy and the lifetime values read out as intensity. The graphs in Figure 5 were plotted using Prism Graph Pad 4 (GraphPad Software, CA, USA), with each dot representing one individual fish. After testing for normality, data was analysed using a two‐way ANOVA with Bonferroni's post test in GraphPad Prism.

## 4 Conclusions

We have successfully generated a novel transgenic zebrafish line expressing a Caspase 3 FRET biosensor under the control of a ubiquitous promoter in a non‐pigmented zebrafish. We have shown that this can be used for FLIM and FRET experiments implemented with OPT to non‐invasively image live organisms. The application of FLIM OPT as a technique for *in vivo* FRET readouts enables visualisation of enzyme activation throughout live intact zebrafish embryos. Here only 150 s were required to acquire the 3‐D FLIM dataset, compared to 300 s required to image a single optical section using LSCM with TCSPC, potentially imposing significantly less stress on the organism. The low light dose associated with wide‐field imaging enables extended time‐lapse studies to be undertaken with the potential to recover the zebrafish after imaging for further longitudinal studies. In this study we applied FLIM OPT to map Caspase 3 activation as a readout of cellular apoptosis initiated by gamma irradiation in 3‐D in zebrafish embryos. To our knowledge, this is the first demonstration of optically visualizing enzyme activation throughout a whole live organism. We believe this approach could be useful in drug discovery, as zebrafish are an ideal model for drug screening due to their high fecundity and transparency to optical radiation 66, and are being utilized more regularly 67, 68. FLIM OPT could provide a way to visualize whole body responses to potential therapies in space and time.

## Supporting information

Supporting Information
