# Supplementary material for: Visualising apoptosis in live zebrafish using fluorescence lifetime imaging with optical projection tomography to map FRET biosensor activity in space and time
Source: J Biophotonics. 2016 Jan 11;9(4):414–24. doi: 10.1002/jbio.201500258 (PMC4858816; doi:10.1002/jbio.201500258)
Supplement: Supplementary file 1 — Supporting Information [file JBIO-9-414-s001.pdf]

Supporting Information

Visualising apoptosis in live zebrafish using fluorescence lifetime imaging with optical projection tomography to map FRET biosensor activity in space and time

Natalie Andrews, Marie-Christine Ramel, Sunil Kumar, Yuriy Alexandrov, Douglas J. Kelly, Sean Warren, Louise Kerry, Nicola Lockwood, Antonina Frolov, Paul Frankel, Laurence Bugeon, James McGinty, Margaret J. Dallman and Paul M. W. French

FLIM of cells expressing SECFP and Caspase 3 biosensor

An in-house built automated FLIM multiwell plate microscope [Supplementary reference[1]] was used to determine the lifetime of SECFP and the uncleaved Caspase 3 FRET probe in live cells.

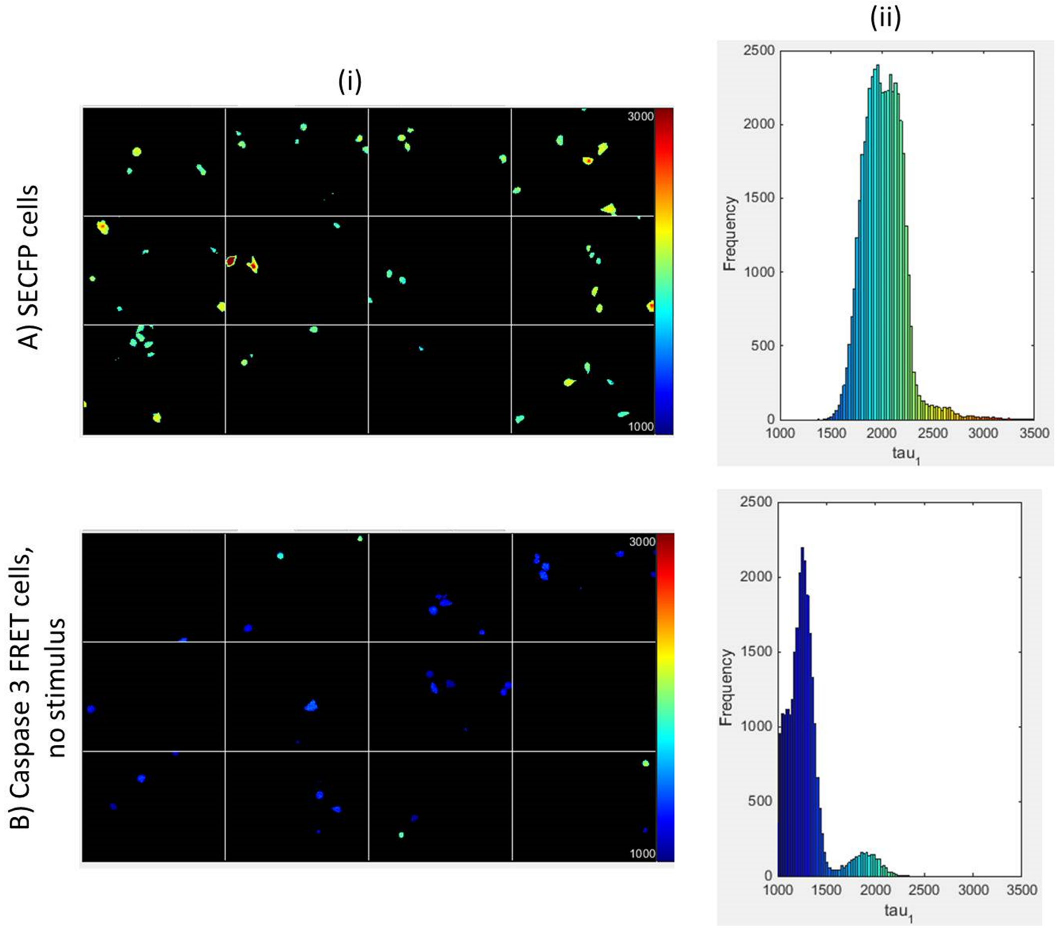

Figure S1. SECFP only and uncleaved FRET sensor analysis to give reference lifetimes. HeLa cells were transfected with SECFP or the caspase 3 FRET probe using Effectene. 24 hours later, the lifetimes of cells in the selected FOVs were acquired. A) SECFP results; B) FRET results; i) False colour scale FLIM lifetime maps generated in *FLIMfit*; ii) corresponding pixel lifetime histograms, generated in *FLIMfit.*

The data presented in **Figure S1** shows a consistent lifetime in SECFP transfected individual cells of around 2 ns and around 1.25 ns in Caspase 3 FRET probe transfected cells. The fit results table generated using *FLIMfit* was exported and the mean lifetime and standard deviation were calculated for each group, and are presented in**Table S1**. These results can be used to ensure accuracy of experiments and data fitting.

Plasmid Transfected	*Mean Lifetime*	*Standard deviation*
SECFP	1.97 ns	0.12
Caspase 3 FRET biosensor	1.33 ns	0.16

Table S1. Mean lifetime and standard deviation of SECFP and FRET probe transfected into HeLa cells.

Materials and methods

Mammalian Cell Culture

Cell lines were maintained in sterile plastic ware (75cm^2 vented flasks, BD Falcon) at 37 °C in a humidified chamber controlled at 5% CO_2. All procedures were carried out aseptically in laminar flow hoods, except preparation of cells for flow cytometry, which was carried out on the bench. Both COS-7 and HeLa cells were cultured in DMEM. THP-1 cells were cultured in RPMI. Cells were passaged using 1x Trypsin-EDTA, as described by ATCC. Cells were stored in 10% DMSO at -80°C for long term storage.

HeLa Effectene Transfection

DNA was used at a concentration of 0.1 µg per well diluted in buffer EC, to give a total volume of 29.2 µL per well. 0.8 µL enhancer was added per well. The mix was incubated at room temperature for 5 minutes. 0.5 µl Effectene reagent was mixed with 19.5 µl buffer EC per well. This was added to the DNA-Enhancer mixture, mixed by pipetting up and down, and then incubated for 20 min at room temperature. 50 µL of the DNA transfection mix was added to wells of a 96-well plate (Greiner). 150 µl cell suspension, was then added and mixed by pipetting up and down twice. Cells were incubated and assayed after 24-48 hours.

Automated multiwell plate FLIM microscope

Cells were plated on 96 well µClear plates (Greiner) and transfected prior imaging. The automated multiwell plate FLIM microscope is built around a wide-field motorised microscope (Olympus IX81-ZDC). A spectrally-filtered ultrafast fiber-laser-pumped super-continuum source (SC-400-6, Fianium Ltd) was used to excite fluorophores. Cells were imaged using a 40x objective long working distance air objective (Olympus, LUCPLFLN

40). An integration time of 0.1 s was used. Gates were acquired every 1000 ps from 1000-9000 ps relative to the delay. The delay was set to 900 ps and gain was to 650 V. Fluorescence emission was detected using a gated optical intensifier (Kentech Instruments; model HRI) read out by a cooled CCD (Hamamatsu Photonics; model Orca ER II).

FLIM of yolk sac

The yolk sac presents a problem during analysis, as it has a short autofluorescence lifetime upon excitation with SECFP wavelengths. In **Figure S2**, the yolk sac has been segmented and analysed. It was shown to have a mean lifetime of 1.17 ns (standard deviation 0.21).

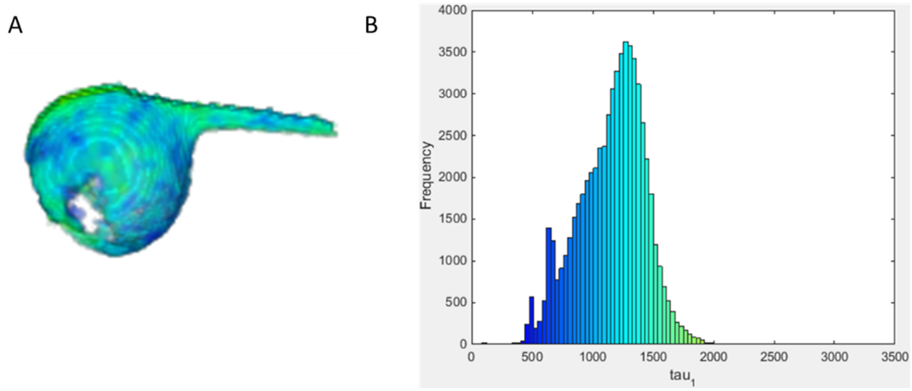

Figure S2. Lifetime analysis of the yolk sac. A) The embryo yolk sac was segmented using Fiji to create a 3D fluorescence lifetime map B) corresponding pixel lifetime histograms, generated in FLIMfit.

Confocal Spectral FRET of Tg(Ubi:Caspase3bios) zebrafish embryos

Tg(Ubi:Caspase3bios) zebrafish embryos at 48 hpf were dechorinated and irradiated with 25 Gy gamma-irradiation. At 3.5 hours post irradiation they were mounted in 0.5% low melt agarose containing 4.2% MS222 and imaged using a confocal SP5 microscope alongside a non-irradiated zebrafish control group at similar hpf. These spectral FRET experiments were undertaken starting with embryos at 48 hpf due to their relatively high brightness and the low sensitivity of the detectors available. Images were acquired in the donor channel (462-485 nm) and acceptor channel (525-550 nm).

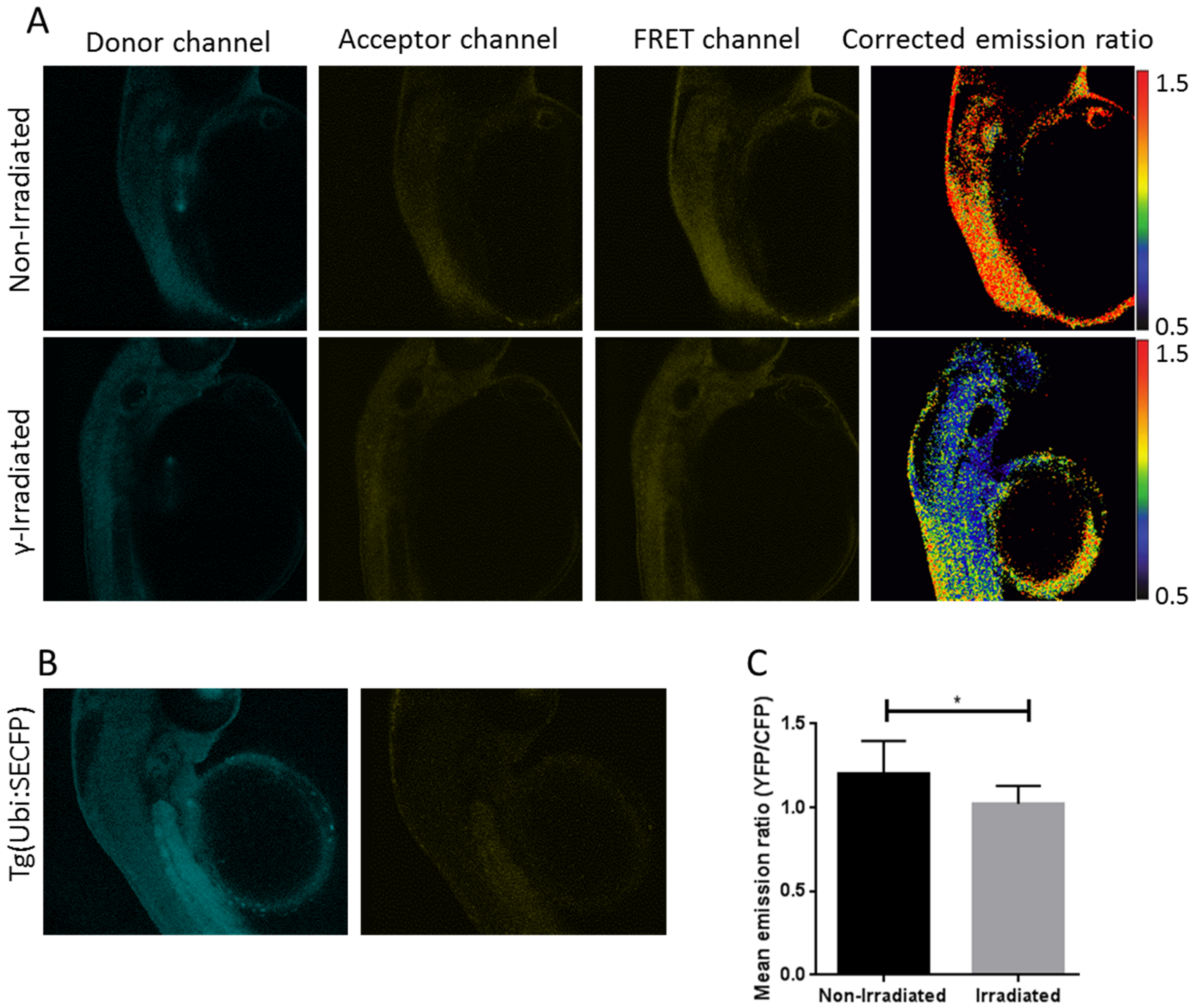

Figure S3 A) Spectrally resolved fluorescence images of 48 hpf Tg(Ubi:Caspase3 biosensor) embryos: "Donor channel" corresponds to donor excitation & donor channel detection; "Acceptor channel" corresponds to acceptor excitation & acceptor channel detection; "FRET channel" corresponds to donor excitation & acceptor channel detection and "Corrected emission ratio" corresponds to the corrected mean acceptor/donor emission ratio calculated using FiJi following the protocol published by Kardash et al [27]. B) 48 hpf Tg(Ubi:SECFP) embryo (i.e. expressing only the donor fluorophore) showing cross-talk in acceptor channel. C) Graph showing a statistical significance of $p>0.05$ (*) between corrected mean acceptor/donor emission ratio of irradiated versus non-irradiated embryos. This corrected acceptor/donor emission ratio was averaged over all the pixels in each field of view and statistical analysis across multiple experiments was performed with $n\geq7$ in each category using GraphPad Prism 6. Data showed a normal distribution so an unpaired t-test was used.

FLIM OPT of Tg(Ubi:SECFP) zebrafish embryos

To confirm the changes seen in the Tg(Ubi:Caspase3bios) zebrafish embryos were due to irradiation treatment, donor–only Tg(Ubi:SECFP) fish were irradiated and imaged using FLIM OPT.

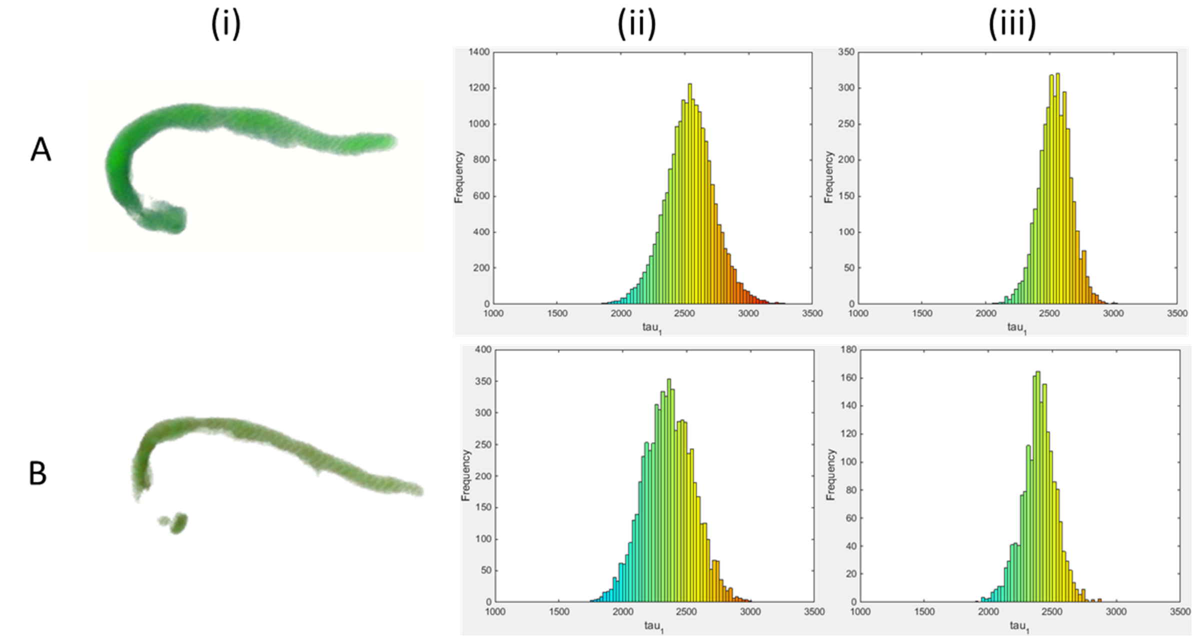

Figure S4: 24 hpf Tg(Ubi:SECFP) zebrafish embryos were irradiated with 25 Gy and imaged at 3.5 hours post irradiation. Data was fitted using *FLIMfit*. i) False colour intensity merged lifetime map of Tg(Ubi:SECFP): A) irradiated, B) non-irradiated; ii-iii) corresponding pixel lifetime histograms, generated in FLIMfit: ii) segmented head, iii) segmented tail.

The histograms and intensity merged images displayed in figure S4 for both the irradiated and non-irradiated Tg(Ubi:SECFP) zebrafish show that the lifetime over the whole of the specimens is close to ~2 ns, i.e. similar to the lifetime of SECFP expressed alone in cells, as shown in supplementary **figure S1**. This indicates that the significant changes in lifetime observed in the irradiated Tg(Ubi:Caspase3bios) zebrafish embryos are due to a change in the Caspase 3 FRET biosensor rather than changes in the donor fluorophore environment.

Generating segmentation masks

To obtain segmentation masks, the brightest (3000 ps) and dimmest (7000 ps) time gates were used, as the dimmest gate does not show the yolk sac, due to its fast fluorescence decay. First, the background B was estimated as being beyond fixed quantile q=0.9 of the image's global intensity histogram. The embryo was segmented by the thresholding above the value T = median(B) + K*std(B), where K=4. The segmented image was then refined by the

morphological hole filling and the radius 2 closing operations. In the next step, all segmented objects were removed except biggest one (the body of the embryo). Finally, the remnants of the yolk sac envelope were removed by the morphological opening with the radius 7. It was found that these settings were suitable for most images. This allowed fast batch processing of the data. These segmentation masks could then be applied to the images in FLIMfit prior to data analysis.

Supplementary References

[1] Automated fluorescence lifetime imaging plate reader and its application to Förster resonant energy transfer readout of Gag protein aggregation, D. Alibhai, D. J. Kelly, S. Warren, S. Kumar, A. Margineanu, R. A. Serwa, E. Thinon, Y. Alexandrov, E. J. Murray, F. Stuhmeier, E. W. Tate, M. A.A. Neil, C. Dunsby and P. M.W. French, J. Biophotonics 6 (2012) 398-408, doi: 10.1002/jbio.201200185
